# Sex differences in the pre-hospital ambulance delay, assessment and treatment of patients with acute coronary syndrome: a rapid evidence review

**DOI:** 10.29045/14784726.2024.3.8.4.21

**Published:** 2024-03-01

**Authors:** Holly de Banke Munday, Gregory Adam Whitley

**Affiliations:** University of Lincoln; University of Lincoln; East Midlands Ambulance Service NHS Trust ORCID iD: https://orcid.org/0000-0003-2586-6815

**Keywords:** acute coronary syndrome, emergency medical services, healthcare disparities, paramedicine, sex characteristics

## Abstract

**Background:**

Chest pain is a frequent symptom suffered by adult patients attended by ambulance. Evidence suggests female patients may suffer different symptoms to their male counterparts, potentiating differences in pre-hospital time delays, assessment and treatment.

**Objective:**

To explore the sex differences in the pre-hospital ambulance delay, assessment and treatment of patients with acute coronary syndrome (ACS).

**Methods:**

A rapid evidence review was conducted following the Cochrane rapid review guidelines. MEDLINE and CINAHL Complete were searched via EBSCOhost on 2 February 2023, and reference lists of included studies and reviews were screened. The Joanna Briggs Institute checklist for analytical cross-sectional studies was used to perform critical appraisal, and a narrative synthesis was conducted.

**Results:**

From 216 articles screened, nine were included, representing over 3.1 million patients from five different countries. Female patients were more likely to suffer delays in time to first electrocardiogram (ECG) and delays in transport time to the emergency department by ambulance. Female patients were also less likely to receive an ECG, aspirin, glyceryl trinitrate and other analgesics.

**Conclusion:**

There are sex disparities in the pre-hospital ambulance delay, assessment and treatment of patients with ACS. Future research is urgently needed to fully understand the reasons for these observations.

## Introduction

Acute coronary syndrome (ACS), defined as unstable angina or acute myocardial infarction (including electrocardiographic ST-segment elevation myocardial infarction (STEMI) and non-ST-elevation myocardial infarction (NSTEMI)) (Gale, 2017), is a primary cause of mortality. ACS was the leading cause of death in 2015 for men, and the second leading cause for women (National Institute for Health and Care Excellence, 2020). Acute chest pain, which is the most common symptom of ACS in both men and women (Singh et al., 2022), accounts for more than one in six emergency ambulance transports to hospital and one in 20 of all presentations to the emergency department (Pedersen et al., 2019).

Despite this vast number of presentations, there is a growing amount of evidence to suggest sex differences in the management and ambulance delay of patients with ACS (Dawson et al., 2023). Although the reason for this is mostly unclear, it is known that there is deep-rooted sexism and misogyny within healthcare and medicine (British Medical Association, 2021; Prakash et al., 1993; Stone et al., 2020); until as recently as the last decade, women were excluded from many biomedical trials (Johnson et al., 2003; Liu & DiPietro Mager, 2016) due to the assumption that the findings from men can be generalised to women (Miller & Kollauf, 2002).

Sex refers to the set of biological attributes that are associated with physical and physiological features, such as chromosomal genotypes and hormonal levels, whereas gender generally refers to socially constructed roles, behaviours and identities of women, men and gender-diverse people that occur in a historical and cultural context and may vary across societies and over time (Elsevier, 2023). For the purpose of this review, disparity in care will be discussed in the context of patient sex, and the terms ‘female’ and ‘woman’ are considered synonymous.

In relation to ACS, it is often taught that women have atypical symptoms (Ramamoorthy et al., 2021), when in fact the symptoms just differ from those experienced by men (Sella et al., 2021; van Oosterhout et al., 2020). Due to this, the aim of this review is to conclude whether there are sex differences in the pre-hospital ambulance delay, assessment and treatment of patients with ACS.

## Methods

### Study design

A rapid evidence review was conducted following the Cochrane rapid review guidance (Garritty et al., 2020).

### Search strategy

The PICo (population, phenomena of interest, context) qualitative framework was used to develop the following research question (Siriwardena & Whitley, 2022): *are there sex differences in the pre-hospital ambulance delay, assessment and treatment of patients with ACS?*

The MEDLINE and CINAHL Complete databases were searched simultaneously, using EBSCOhost to find appropriate literature. The following keywords and phrases were used: (gender OR sex) AND (difference or bias) AND (“acute coronary syndrome” OR “chest pain” OR “myocardial infarction”) AND (paramedic OR ambulance OR prehospital).

The following limitations were placed on the search.

Inclusion criteria:

- papers reported in English;
- pre-hospital ambulance setting;
- date range of 1970 to 2 February 2023;
- published in academic journals; and
- reference to sex or gender differences and ACS.

Exclusion criteria:

- protocols, abstracts, opinion pieces.

Reference list screening and content experts were also used to find additional papers.

### Study screening and data extraction process

Following the Cochrane rapid review guidance (Garritty et al., 2020), after the first advanced search with multiple results, the above databases, inclusion and exclusion criteria were used to perform a title and abstract screen on the identified studies. After this, the full-text articles were retrieved and screened. Screening was performed by one author (HdBM) using Microsoft Excel. The included studies were illustrated within a table.

### Critical appraisal

The included studies were critically appraised by one author (HdBM) following the Joanna Briggs Institute (2020) analytical cross-sectional studies checklist. The results were displayed in tabular format. Risk of bias was not used as a reason for inclusion; instead, findings informed by studies at high risk of bias were interpreted with caution.

### Synthesis

A narrative synthesis was used to synthesise the data included in this review (Popay et al., 2006). A narrative synthesis is a way to get a first systematic look at the data and start analysing them to explain the findings (Popay et al., 2006), and although it can involve the manipulation of statistical data it normally adopts a textual approach (Thomson & Campbell, 2020). The narrative synthesis was conducted by one author (HdBM) and discussed iteratively with the other (GAW). Some of the statistics were extracted directly from the included studies, whereas some were collated and produced by the author.

Synthesis was considered deductive using the following pre-determined themes:

- ambulance delay time;
- assessments of patients; and
- treatment of patients.

The statistics used within the narrative synthesis were displayed in tabular format.

### Reflexivity

Reflexivity is defined as when a researcher acknowledges their own bias and beliefs, and therefore must self-consciously critique and appraise how their subjectivity and context influence their research processes (Olmos-Vega et al., 2022). HdBM acknowledges the concept of feminist ideology and how this can influence the review since feminist methodology proposes that women have different health experiences to those traditionally cultivated and valued by medical science (Ruzek et al., 1997). Despite this, the author is keen to display an unbiased viewpoint and look solely at the evidence; however, due to HdBM’s own feminist stance and the fact that most of this review was undertaken independently by HdBM, this could be challenging.

## Results

**Figure 1:**
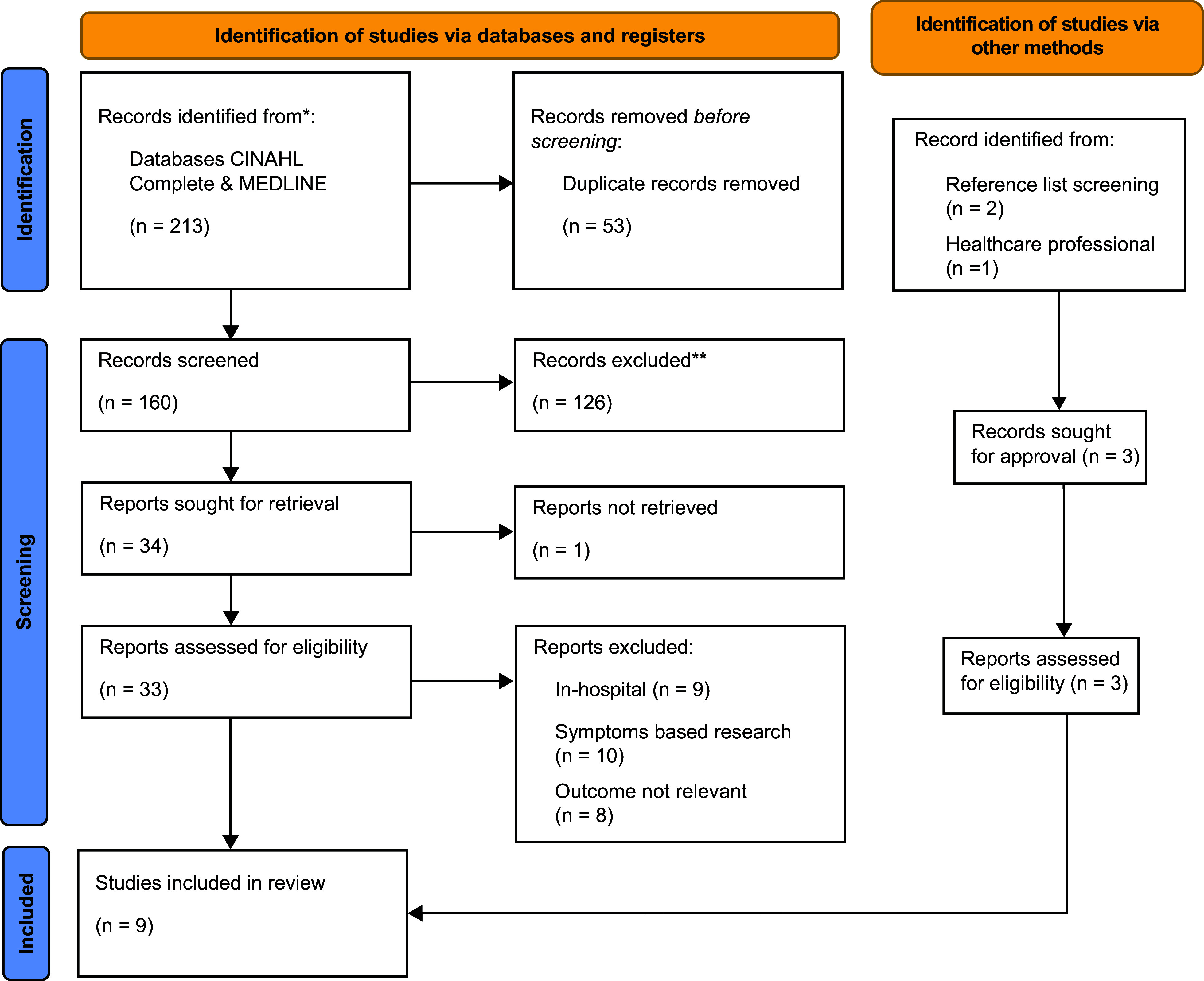
Figure 1. PRISMA flow diagram (Page et al., 2021).

After 216 studies were screened and 36 studies were sought for retrieval from both databases and other methods, nine studies were selected for inclusion. The screening process was recorded within a PRISMA flow diagram (see Figure 1).

Nine studies were included, representing 3,186,270 patients from five countries: United States, Australia, Norway, Sweden and Ireland. No clinical trials were found, and all included studies were observational (see Table 1).

**Table 1.** Summary of included studies.

Author and date	Design type	Country and number of participants	Study aim	Results
Banks & Dracup (2007)	Quantitative observational study	USA 61	To identify gender differences in delay time and the reasons why African Americans delay in seeking medical care for the symptoms of acute MI.	Median delay time was 4.4 hours for women and 3.5 hours for men, although the difference was not significant. Women who were alone when symptoms began delayed longer than women who were with someone.
Dawson et al. (2023)	Quantitative observational study	Australia 256,901	To assess sex differences in epidemiology and care pathways from EMS contact through to clinical outcomes following discharge.	Women were less likely to receive guideline-directed care for transport to hospital, pre-hospital aspirin or analgesia administration, 12-lead ECG, IV cannula insertion and offload from EMS within target times. They were also less likely to undergo angiography or be admitted to cardiac unit. Long-term and 30-day mortality was also higher for women.
Hsu et al. (2021)	Quantitative observational study	Australia 110,044	To examine sex differences in the pre-hospital EMS care of patients hospitalised with MI.	Women were less likely than men to be assessed as having ACS (23% vs 28%). Women had 23% lower odds than men of receiving the MI protocol, 21% lower odds of receiving aspirin, 16% lower odds of receiving GTN and also 12% lower odds than men of having ECG performed.
Lewis et al. (2019)	Quantitative observational study	USA 2,814,041	To determine whether gender disparities exist in the pre-hospital management of chest pain or OHCA among patients who accessed the EMS.	Women with chest pain received a lower percentage of recommended treatments than men. Women were significantly less likely to be transferred using lights and sirens and they received fewer aspirin treatments than men.
Meisel et al. (2010)	Quantitative observational study	USA 683	To investigate the relationship between sex and the out-of-hospital treatment of patients with chest pain.	Women were less likely than men to receive aspirin, GTN or an IV. They were also less likely to receive treatments among the small sub-group of patients who were later diagnosed with MI.
Melberg et al. (2013)	Quantitative observational study	Norway 244	To understand why women with ST elevation tend to have longer treatment delays than men.	More women than men reported chest pain and discomfort in other areas of their body. Longer patient delays and system delays led to longer ischemic time in women. Women had a lower priority for an ambulance despite similar symptoms to men.
Muhrbeck et al. (2020)	Quantitative observational study	Sweden 539	To investigate the proportion of STEMI patients with a pre-hospital ECG within 10 minutes of ambulance arrival.	A pre-hospital ECG was obtained within 10 minutes for 29% of male patients and 14% of women patients. Women had a two-minute longer delay between ambulance arrival and ECG than men. They also had significantly longer patient delay.
O’Donnell et al. (2006)	Quantitative observational study	Ireland 890	To report the findings of a study that identified gender-specific pre-hospital care pathway delays amongst Irish women and men with MI.	Women are more likely to experience prolonged initial symptom onset to A&E delays and intense symptom onset to A&E delays. Advancing age, public patients and patients who arrived by any other admission route than driving themselves had pre-hospital delays.
Rothrock et al. (2001)	Quantitative observational study	USA 2858	To determine whether there is a gender bias in the pre-hospital management of patients with acute chest pain.	Male patients were more likely to receive aspirin and 12-lead ECGs compared to female patients. However, transport refusal, oxygen, nitro-glycerine and narcotic administration did not differ.

A&E: accident and emergency; ACS: acute coronary syndrome; ECG: electrocardiogram; EMS: emergency medical services; GTN: glyceryl trinitrate; IV: intravenous; MI: myocardial infarction; OHCA: out-of-hospital cardiac arrest; STEMI: ST-segment elevation myocardial infarction.

**Figure 2:**
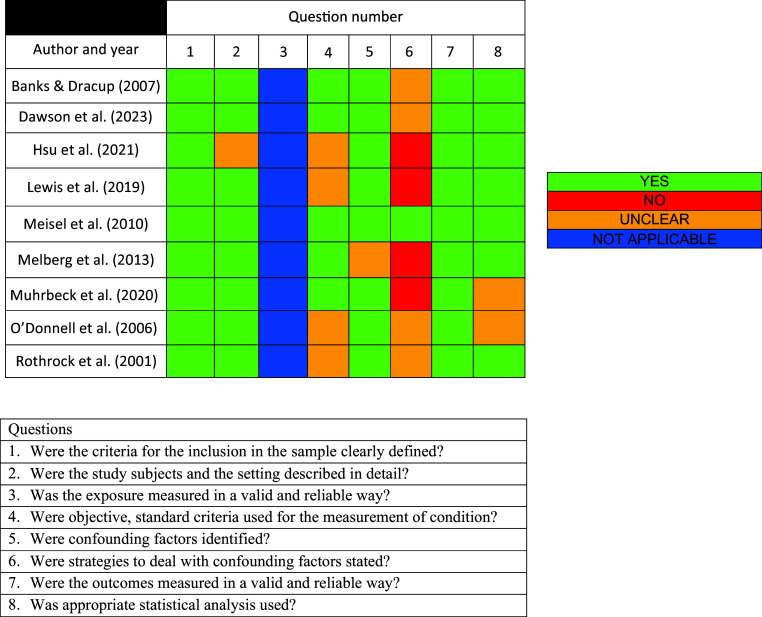
Figure 2. Critical appraisal assessment.

The critical appraisal results showed a moderate risk of bias across studies, with strategies to deal with confounding factors unaddressed by most study authors. We were unable to mitigate this moderate risk of bias using statistical techniques, which reduces the strength of our findings and recommendations. See Figure 2 for the results of the critical appraisal assessment.

The findings of the included studies were tabulated to display the quantitative data split into each theme. See Table 2 for the synthesis table.

**Table 2.** Synthesis table.

	Study (number of participants)								
Predictors	Hsu et al. (2021) (n = 110,044)	Lewis et al. (2019) (n = 2,814,041)	Meisel et al. (2010) (n = 683)	Rothrock et al. (2001) (n = 2858)	Dawson et al. (2023) (n = 256,901)	Muhrbeck et al. (2020) (n = 539)	Melberg et al. (2013) (n = 244)	Banks & Dracup (2007) (n = 61)	O’Donnell et al. (2006) (n = 890)
Patient sex									
Male	70,495	1,153,769	342	1350	127,805	385	179	32	613
Female	39,549	1,223,754	341	1508	129,096	154	65	29	277
Ambulance response times for women (compared to men)					OR: 0.99 95% CI: 0.98–1.01	14 minutes compared to 13 minutes p-value = 0.084	57 minutes compared to 35 minutes p-value = 0.006		
Symptom to ED time for women taken via ambulance (compared to men)								3.1 hours compared to 0.58 hours p-value = 0.04	14 hours compared to 2.8 hours p-value = < 0.0001
Ambulance arrival to ECG in 10 minutes for women (compared to men)						99 minutes compared to 19 minutes p-value = 0.001			
Women receiving ECG assessment (compared to men)	OR: 0.88 CI: 0.83–0.92 12% less likely than men	48.8% compared to 50.5% Difference: -2.7% 95% CI: -5.7, 2.2	OR: 0.80 79% compared to 82% p-value = 0.21	OR: 0.50 39.3% compared 46.8% p-value = < 0.001	OR: 0.82 95% CI: 0.81–0.83				
Women receiving aspirin (compared to men)	OR: 0.79 CI: 0.76–0.81 21% less likely than men	42.5% compared to 45.3% Difference: -2.8% 95% CI: -4.8, -0.8	OR: 0.60 24% compared to 32% p-value = 0.03	OR: 0.71 35.4% compared to 42.3% p-value = < 0.001	OR: 0.86 95% CI: 0.84–0.87				
Women receiving GTN (compared to men)	OR: 0.84 CI: 0.81–0.86 16% less likely than men	41% compared to 43.7% Difference: -2.7% 95% CI: -4.8, -0.6	OR: 0.60 26% compared to 33% p-value = 0.02	OR: 1.0 32.9% compared to 33.1% p-value = 0.569					
Women receiving analgesic administration for pain (compared to men)					OR: 0.85 95% CI: 0.83–0.88				

### Ambulance delays

Women and men received similar response times from the ambulance service: Muhrbeck et al. (2020) found that women had to wait on average only one more minute than men for an ambulance to arrive (p = 0.084), and Dawson et al. (2023) also found that women and men received a similar response time: odds ratio (OR) 0.99 (95% confidence interval (CI) 0.98–1.01). Melberg et al. (2013) found that women wait significantly longer for an ambulance, as they found the average waiting time for females was 57 minutes compared to 35 minutes for men, however this study had a much smaller sample size (see Table 2).

Banks and Dracup (2007) and O’Donnell et al. (2006) both found that the time from symptom onset to A&E via an ambulance was considerably greater for women than men, with O’Donnell et al. (2006) finding women waited 14 hours, whereas men waited just 2.8 hours.

Muhrbeck et al. (2020) found that the time from ambulance arrival to electrocardiogram (ECG) was 99 minutes for female patients, compared to 19 minutes for male patients (p = 0.001).

### Assessments of patients

Three studies found that women were statistically less likely to receive an ECG (Dawson et al., 2023; Hsu et al., 2021; Rothrock et al., 2001), and two studies found that women were less likely to receive an ECG but the difference was not statistically significant (Lewis et al., 2019; Meisel et al., 2010). Dawson et al. (2023) found that women were less likely to receive an ECG than men: OR 0.82 (95% CI 0.81–0.83). Hsu et al. (2021) found that women were 12% less likely to receive an ECG compared to men, with an OR of 0.88 (95% CI 0.83–0.92). Rothrock et al. (2001) found that women were less likely to receive an ECG (OR 0.50, p < 0.001). Lewis et al. (2019) found that 48.8% of females received an ECG, whereas 50.5% of men received an ECG, and Meisel et al. (2010) found that fewer women received ECG assessment but the difference was not statistically significant.

### Treatment of patients

Meisel et al. (2010) found that only 24% of women were given aspirin as part of their treatment, compared to 32% of men (p = 0.03). Rothrock et al. (2001) found similar statistically significant results to this, as they found that women were less likely to receive aspirin than men (OR 0.71, p < 0.001). Hsu et al. (2021) and Lewis et al. (2019) also reflect the above results, as they found that men were more likely to receive aspirin than women. Dawson et al. (2023) found an OR of 0.86, with a 95% CI of 0.84–0.87, showing that the odds of female patients receiving aspirin is significantly less than that of male patients.

In relation to patients receiving glyceryl trinitrate (GTN), Hsu et al. (2021), Lewis et al. (2019) and Meisel et al. (2010) all found that women were less likely to receive GTN than men (see Table 2). Rothrock et al. (2001) found that although women were less likely to receive GTN than men, this difference was not significant (OR 1.0, p = 0.569).

Dawson et al. (2023) found that women were less likely to receive analgesic treatment than men (OR 0.85, 95% CI 0.83–0.88).

The result of the quantitative synthesis suggests that women may suffer a longer ambulance delay time and are less likely to receive the correct assessment and treatment for ACS symptoms compared to men.

## Discussion

This review has found that female patients suffering ACS symptoms are more likely to receive suboptimal care when presenting to emergency ambulance services compared to men, specifically in terms of pre-hospital delay, assessment and treatment.

A systematic review explored pre-hospital delay in depth, and found that women were more likely to arrive at the hospital later than men due to several factors, including sociodemographic, medical history and clinical characteristics according to sex (Nguyen et al., 2010). This review builds on the work of Nguyen et al. (2010) by focusing on ACS diagnoses, rather than myocardial infarction alone, and by exploring pre-hospital assessment and treatment of ACS.

Dawson et al. (2023) explored in-hospital care quality and process measures, in addition to pre-hospital measures. They found that women were less likely to be reviewed by a clinician within the target time, less likely to be off-loaded from ambulances within 40 minutes and less likely to receive angiography. These results were strengthened by Lee et al. (2021) and Poon et al. (2012), who also found that women were less likely to receive angiography and percutaneous coronary intervention than men. Women are less likely to receive medications in hospital, such as aspirin and other evidence-based acute treatments for ACS, including dual antiplatelet therapy, heparins and reperfusion therapy for STEMI (Hao et al., 2019; Khesroh et al., 2017).

Unconscious and implicit bias is one of the leading causes of health disparity (Meidert et al., 2023). This can be seen from women’s cancer diagnoses, where patients experience gender inequality from symptom onset to diagnosis (Din et al., 2015), to pain management, where healthcare professionals continuously fail to take pain reported by females seriously by not prescribing medicines that they would to their male counterparts (Zhang et al., 2021). In relation to cardiac-specific disparities, one possible explanation could be due to male clinician fear of female chest examination. This disparity is evident in out-of-hospital cardiac arrest literature, where female patients are less likely to receive bystander cardiopulmonary resuscitation (CPR) or defibrillation (Ahn et al., 2012; Blewer et al., 2018; Perman et al., 2019). Another reason people may feel uncomfortable doing CPR is perhaps due to a lack of training (Bongberg, 2020). Bongberg (2020) explains how the lack of diversity in body shape and female biology of CPR training equipment causes a lack of understanding and unpreparedness when bystander CPR is needed, resulting in sex disparities following female cardiac events.

### Strengths and limitations

This review has several strengths, one of these being that the authors followed the detailed process of the Cochrane guidance with figures and tables to reinforce this. This guidance allowed for a good structure throughout and allowed for the authors to screen all studies systematically. The quantitative data used also make the data easier to analyse, making them more consistent, precise and reliable (Mander, 2022).

However, there were some limitations. These include researcher bias and the fact that this review was performed primarily by one author (HdBM), who may have missed key studies during the screening process. Some papers were also not available for the authors and due to the date range used some studies may have been missed, which in turn could have changed the results.

### Recommendations for policy, clinical practice and future research

Policy and clinical guideline developers should be aware of the disparity in care between male and female patients who require ACS management, and should promote equality of care between the sexes.

There are three recommendations for clinical practice:

1. Increase awareness of disparity: Clinical managers should promote the awareness of these disparities via clinical notices, bulletin boards and service newsletters. Increased awareness is the first step to change.
2. Education and training: Individual clinicians should support their own knowledge via their continuing professional development (CPD). This could include participating in CPD events and webinars, or clinicians doing their own research in how to overcome these disparities as well as understanding what these disparities are.
3. Enhanced recognition: Evidence suggests there may be differences in the symptoms suffered by male and female patients suffering ACS, which may potentiate the pre-hospital disparity of care noted in this review. Therefore, further education and training on the potential sex differences in ACS symptoms is recommended.

Further qualitative research is needed to understand reasons for this observed disparity in care.

## Conclusion

This review has found disparities in the pre-hospital ambulance delay, assessment and treatment of male and female patients with ACS. Key recommendations based around increased awareness and further CPD to better understand the reasons for disparity were made. Further training and a change in the education of female ACS symptoms and presentations have been recommended. Future qualitative research is key to understanding the reasons for this observed disparity in care.

## Author contributions

HdBM: conceptualisation, methodology, formal analysis, writing, original draft, writing, review and editing. GAW: methodology, writing, review and editing, supervising. HdBM acts as the guarantor for this article.

## Conflict of interest

GAW is an associate editor of the *BPJ*.

## Funding

None.
